# Supplementary material for: A low-complexity linker as a driver of intra- and intermolecular interactions in DNAJB chaperones
Source: Nat Commun. 2025 May 31;16:5070. doi: 10.1038/s41467-025-60063-2 (PMC12126530; doi:10.1038/s41467-025-60063-2)
Supplement: Supplementary file 1 — Supplementary Information [file 41467_2025_60063_MOESM1_ESM.pdf]

Supplementary Information for

A low-complexity linker as a driver of intra- and intermolecular interactions in DNAJB chaperones

Billy Hobbs[1], Noor Limmer[1], Felipe Ossa[1], Ella Knüpling[1,2], Samuel Lenton[3], Vito Foderà[3], Arnout P. Kalverda[4] and Theodoros K. Karamanos[1,*]

[1]*Department of Life Sciences, Faculty of Natural Sciences, Imperial College London, Exhibition Road, London, SW7 2AZ, UK*

[2]*Current address: The Francis Crick Institute, 1 Midland Road, London, NW1 1AT, UK*

[3]*Department of Pharmacy, University of Copenhagen, Universitetsparken 2, Copenhagen 2100, Denmark*

[4]*Astbury Centre for Structural Molecular Biology, University of Leeds, Woodhouse Lane, Leeds LS2 9JT, UK*

**Correspondence to: t.karamanos@imperial.ac.uk (TKK)*

Contents:

Supplementary Figures 1-14

Supplementary Tables 1, 2

Supplementary References

Supplementary Figures

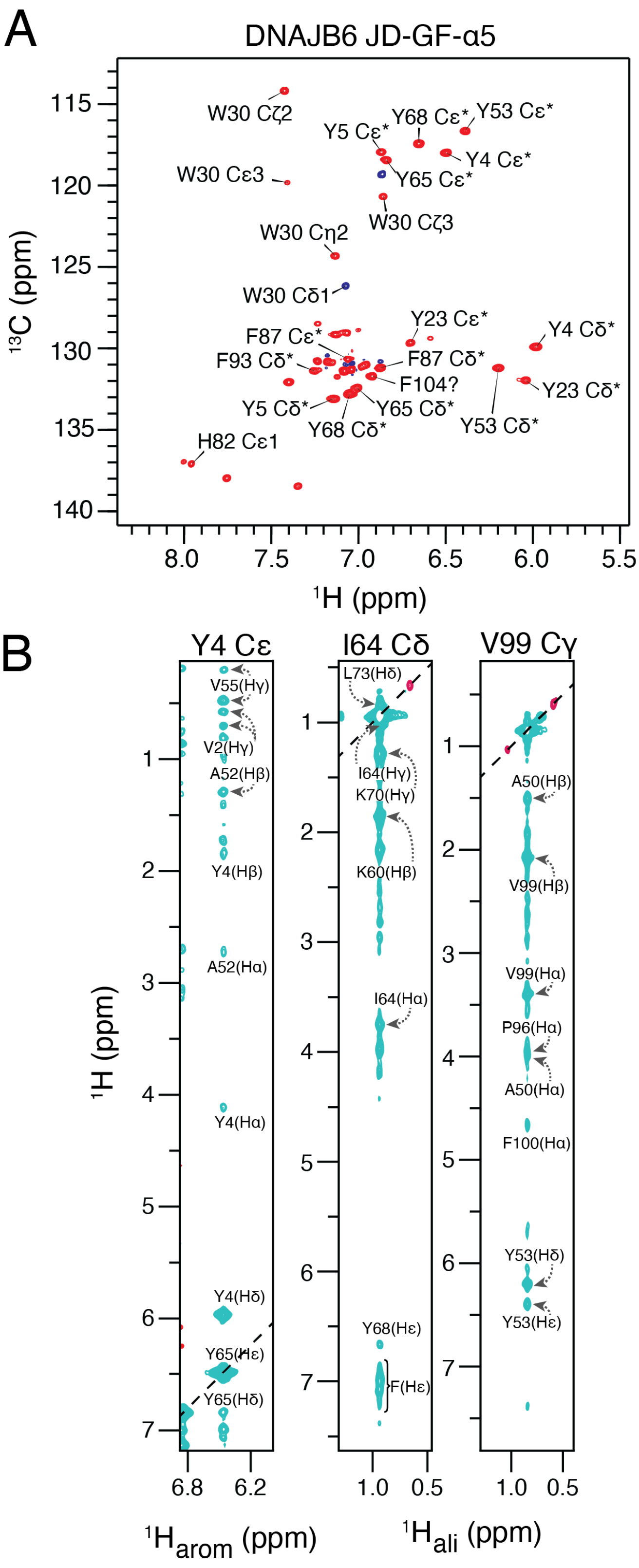

Supplementary Figure 1: NOE analysis of DNAJB6 JD-GF-α5. (A) Assigned aromatic ^{1}H-^{13}C TROSY spectrum collected at 800 MHz. (B) Strips from the 3D aromatic (left panel) or 3D aliphatic (middle and right panels) NOESY-HMQC spectra. Both spectra were collected on a sample of 1 mM ^{13}C, ^{15}N-labelled DNAJB6 JD-GF-α5.

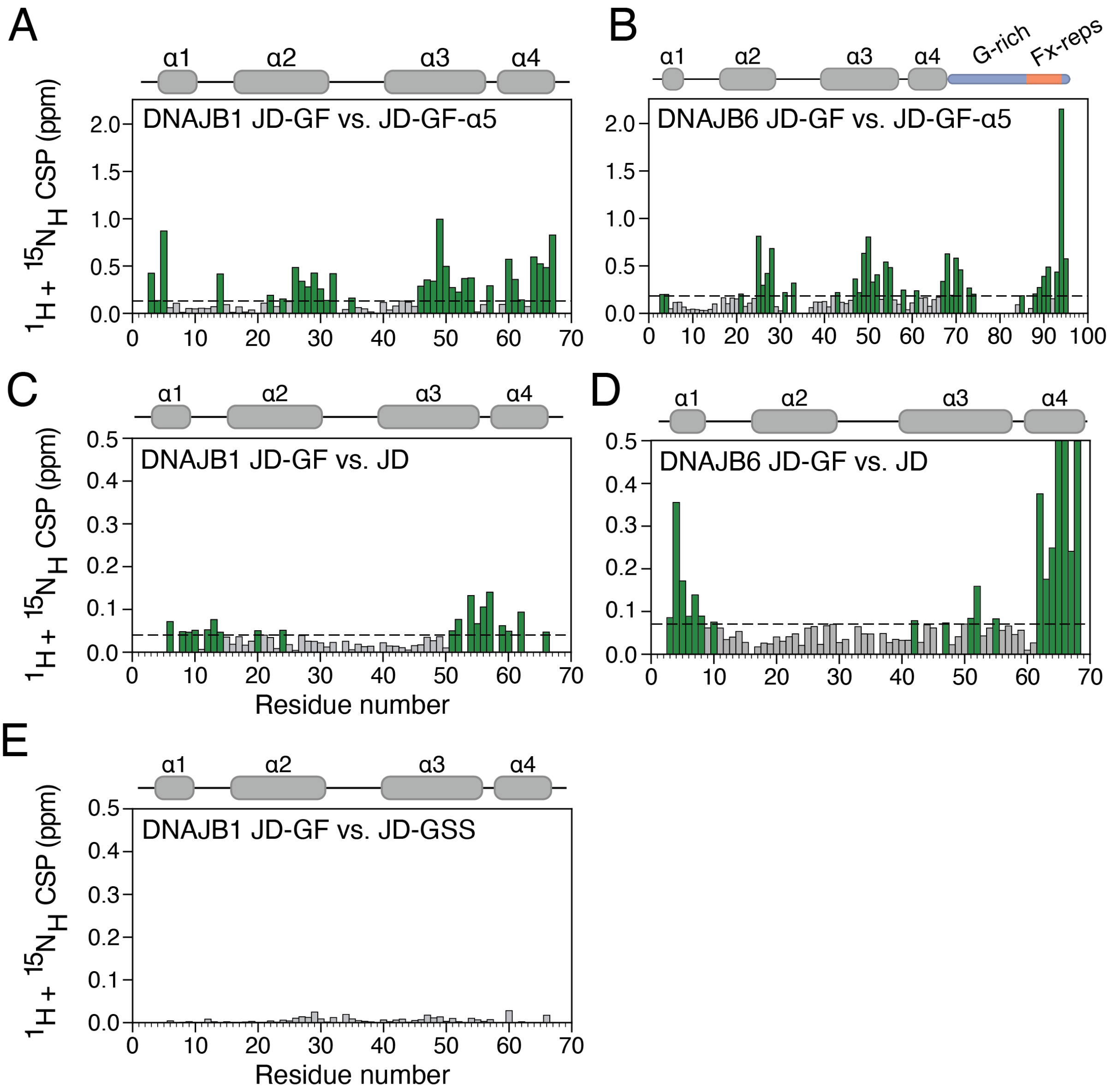

Supplementary Figure 2: Chemical shift analysis of DNAJB6 and DNAJB1 constructs. Combined ^{1}H, ^{15}N chemical shift perturbations between DNAJB1 JD-GF and JD-GF-α5 (A) DNAJB6 JD-GF and JD-GF-α5 (B) or DNAJB1 JD-GF and DNAJB1 JD (C) or DNAJB6 JD-GF and DNAJB6-JD (D) or DNAJB1 JD-GF and DNAJB1 JD-GSS (E). Dashed line shows 2 corrected standard deviations, residues that show CSPs above this cutoff (see Methods) are coloured green. (A) and (B) Highlight differences in the NMR spectra resulting from the release of autoinhibition, while (C) and (D) explore the effect of the native DNAJB6/DNAJB1 linker on the chemical environment of JD. (E) Shows that the native GF of DNAJB1 affects the spectrum of JD in the same manner as a completely disorder GSS linker (compare with Fig. 2A).

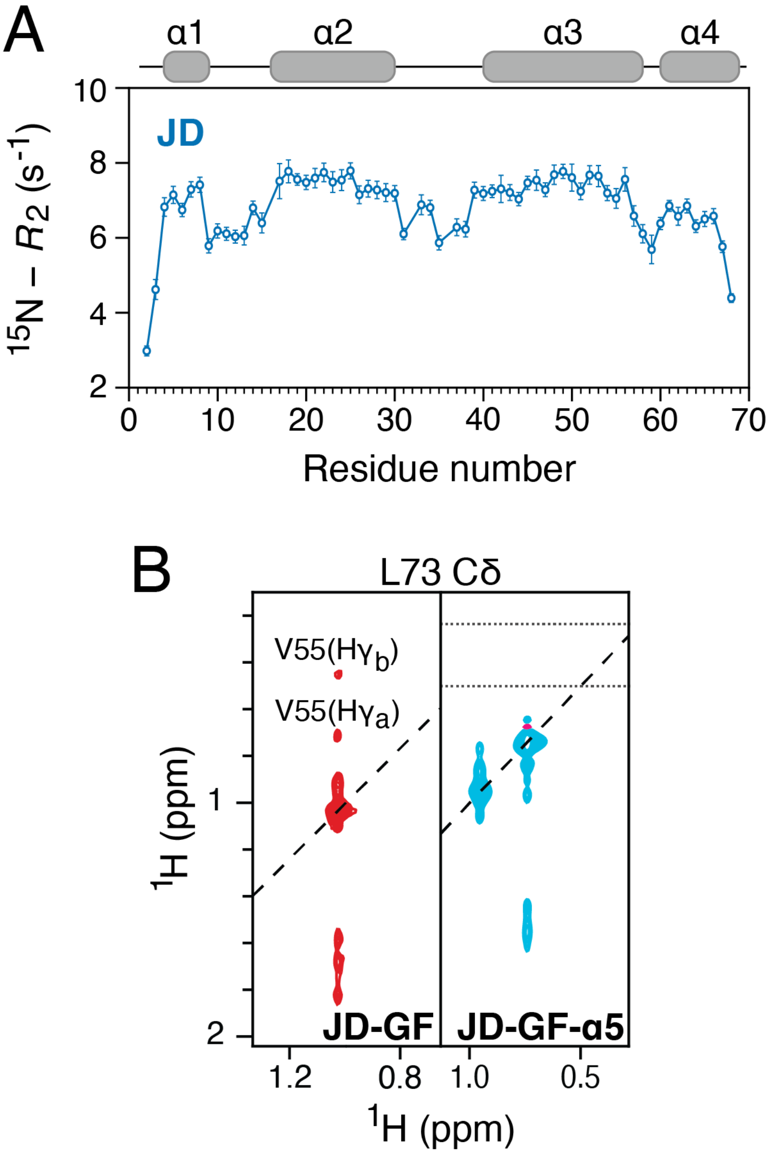

Supplementary Figure 3: ^{15}N relaxation rates for JD alone and comparison on NOEs in JD-GF and JD-GF-α5. (A) ^{15}N-R_2 rates for the isolated JD of DNAJB6 collected on 200 μM ^{15}N-labelled sample at 600 MHz. Error bars represent the errors calculated based on the covariance matrix of a single exponential fit. (B) Strips from the 3D aliphatic NOESY-HMQC spectra for DNAJB6 JD-GF (left panel) and JD-GF-α5 (right panel). For JD-GF-α5 the chemical shift positions for Val55 Hγ protons are indicated with dashed lines. NOEs were observed between the methyl groups of Leu73 and Val55 for JD-GF but not for JD-GF-α5.

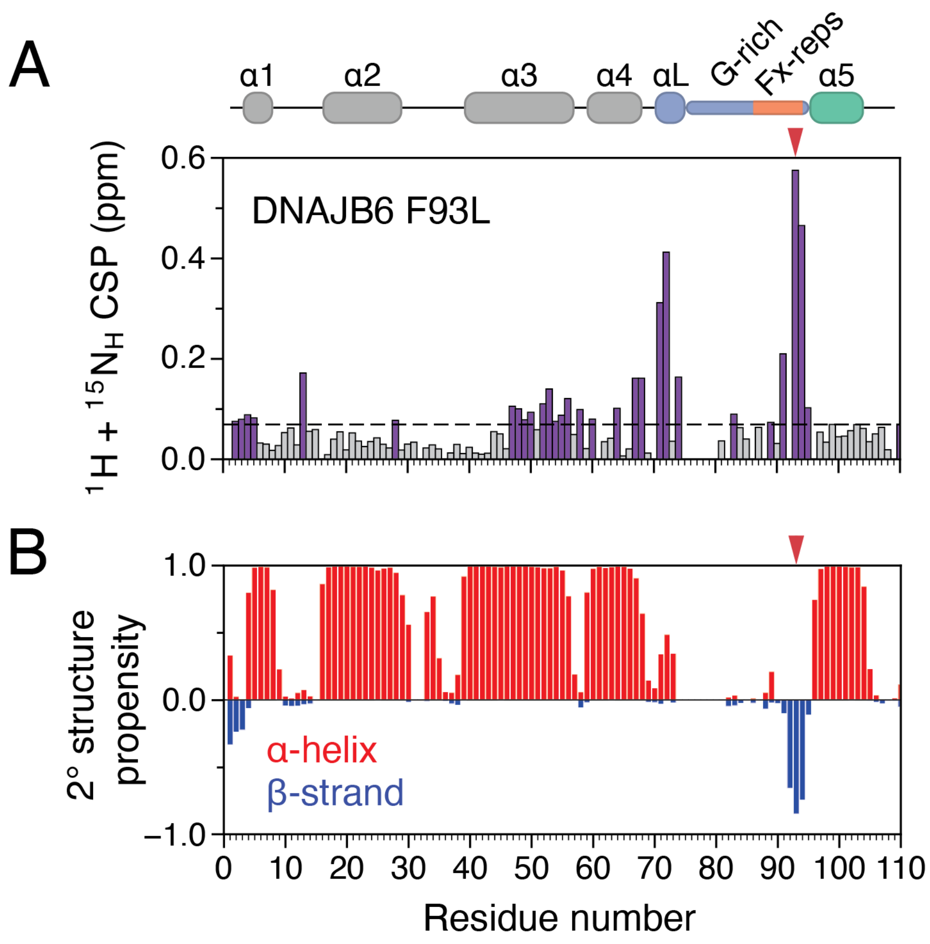

Supplementary Figure 4: The effect of the F93L mutation on DNAJB6. (A) Combined ^{1}H, ^{15}N chemical shift perturbations between wild-type DNAJB6 and F93L. More widespread CSPs are seen for F93L compared to F91L, with some small changes in the J-domain but not in helix 5. Dashed line shows 2 corrected standard deviations, residues that show CSPs above this cutoff (see Methods) are coloured purple. (B) TALOS-computed secondary structure prediction based on the assigned backbone chemical shifts of DNAJB6 F93L. The position of the mutation is marked with an arrow.

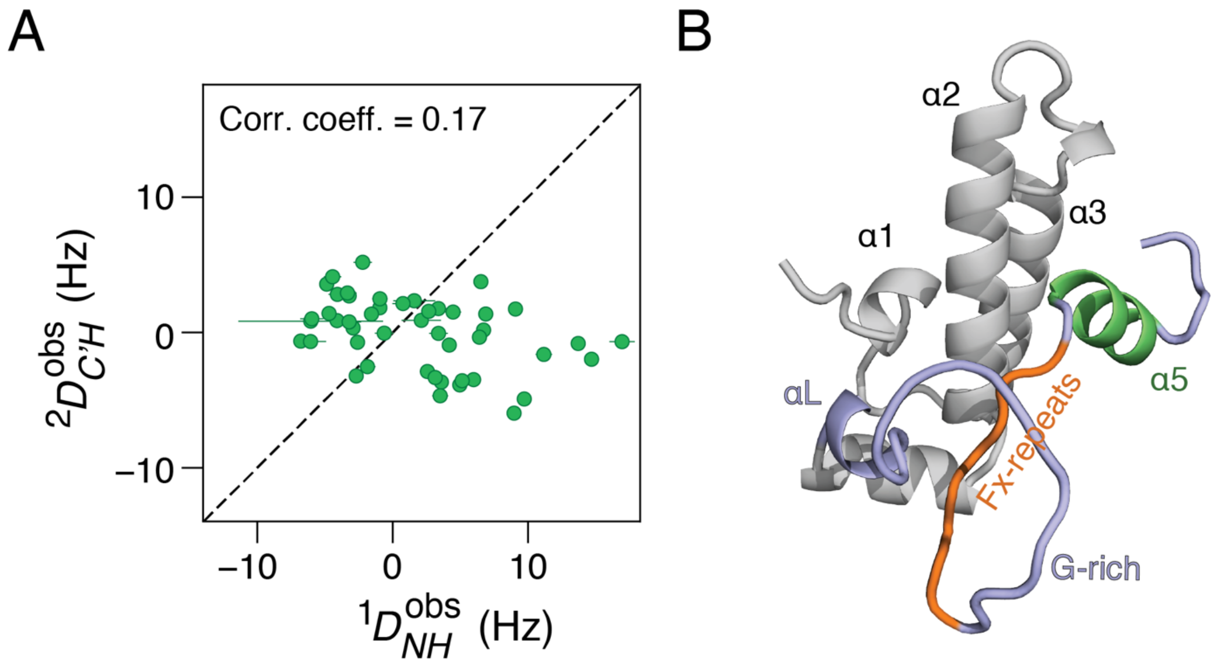

Supplementary Figure 5: RDC analysis on F91L DNAJB6 JD-GF-α5. (A) Correlation between the measured $^{1}D_{\mathrm{NH}}$ and $^{2}D_{\mathrm{C'H}}$ RDC values measured in Pf1 bacteriophage. The two datasets are only weakly correlated (Pearson correlation coefficient of 0.17) providing two almost independent sets of RDCs that can be used to check for agreement with structural models. Error bars were estimated based on the signal to noise ratio of each resonance (B) AlphaFold model of DNAJB6 JD-GF-α5 used to back-calculate the measured RDCs in which residues 70-74 adopt a helical structure (labelled as αL).

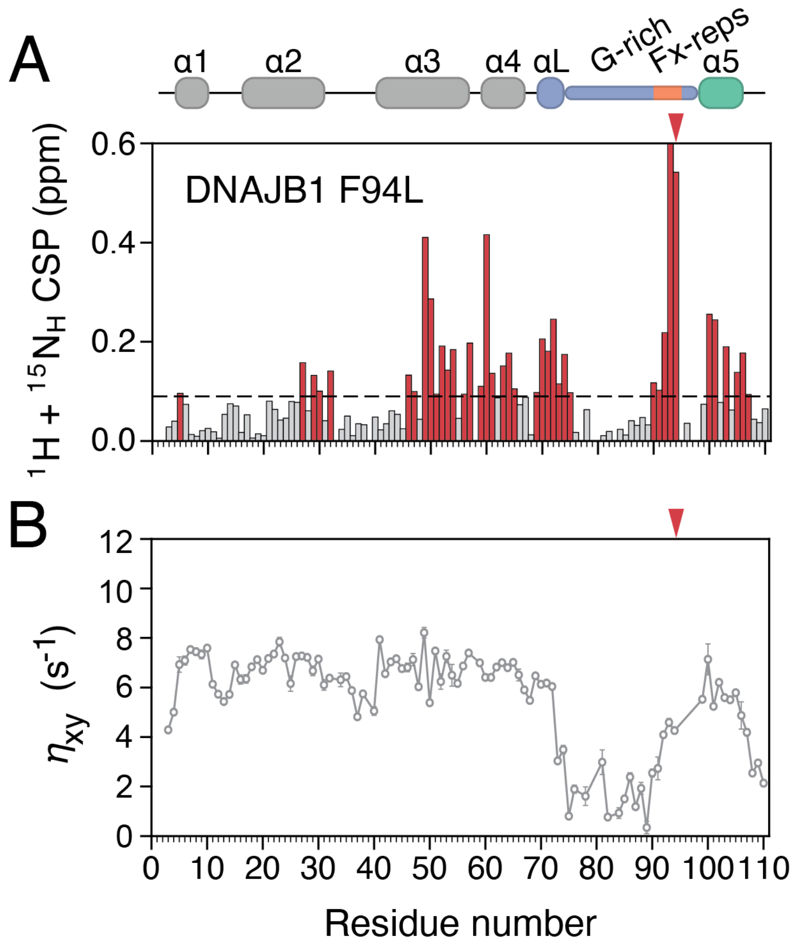

Supplementary Figure 6: The effect of the F94L mutation on DNAJB1. (A) Combined ^{1}H, ^{15}N chemical shift perturbations between wild-type DNAJB1 and F94L JD-GF-α5. Dashed line shows 2 corrected standard deviations, residues that show CSPs above this cutoff (see Methods) are coloured in red. (B) Transverse cross-correlated η_{xy} rates measured at 600 MHz, on 200 µM DNAJB1 F94L JD-GF-α5 using the pulse sequence of Kroenke et al.[1] Error bars represent the errors calculated based on the covariance matrix and the position of the mutation is marked with an arrow.

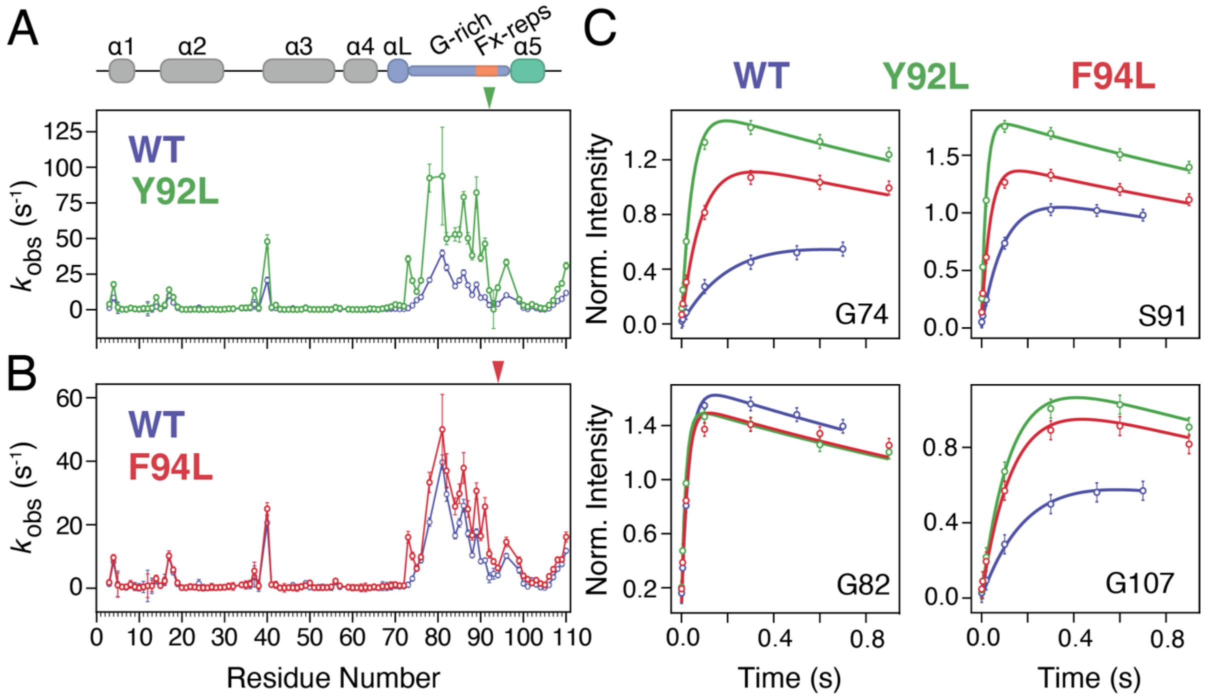

Supplementary Figure 7: Hydrogen exchange data on DNAJB1 variants. (A) Comparison of amide hydrogen exchange rates for WT DNAJB1 JD-GF-α5 and Y92L or F94L (B). (C) Selected raw hydrogen exchange profiles measured with WEX-III pulse sequence.[2] The position of the mutation is marked with an arrow. Error bars in (A) and (B) represent the errors calculated based on the covariance matrix and those in (C) are estimated based on the signal to noise ratio for each resonance.

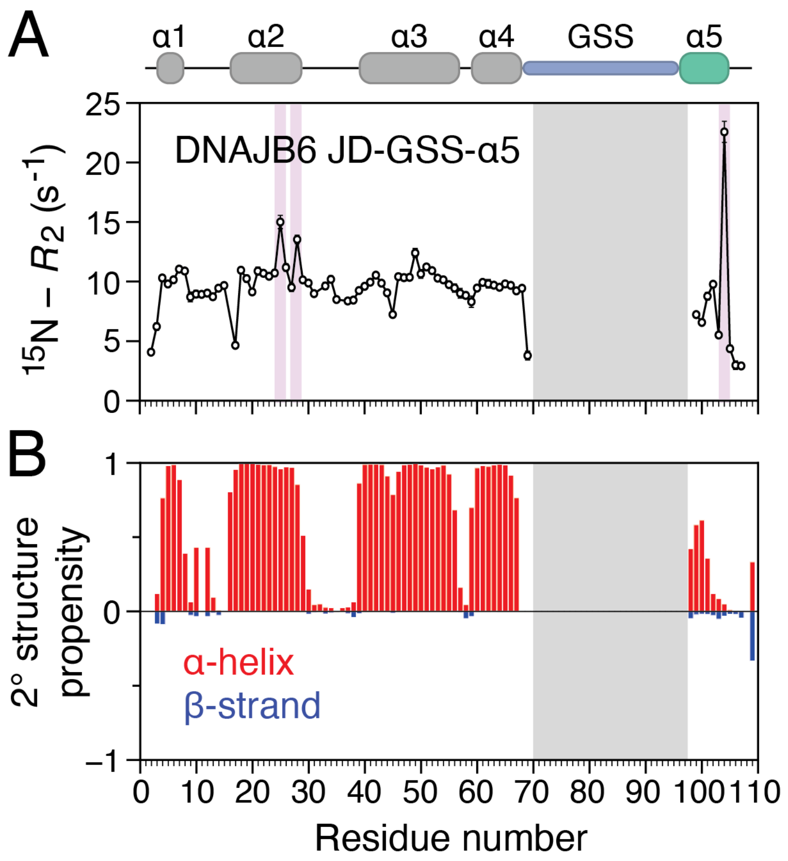

Supplementary Figure 8: Swapping the GF-linker with disordered GSS destabilises DNAJB6's autoinhibition. (A) ^{15}N-R_2 relaxation rates of DNAJB6 JD-GSS-α5 construct. Residues with R_{ex} contributions are highlighted in a purple box. Error bars represent the errors calculated based on the covariance matrix of the single exponential fit. (B) TALOS-computed secondary structure prediction based on the assigned backbone chemical shifts of DNAJB6 JD-GSS-α5 (compare with Supplementary Fig. 4B). The GSS resonances could not be assigned as they completely overlap and are noted with a grey box.

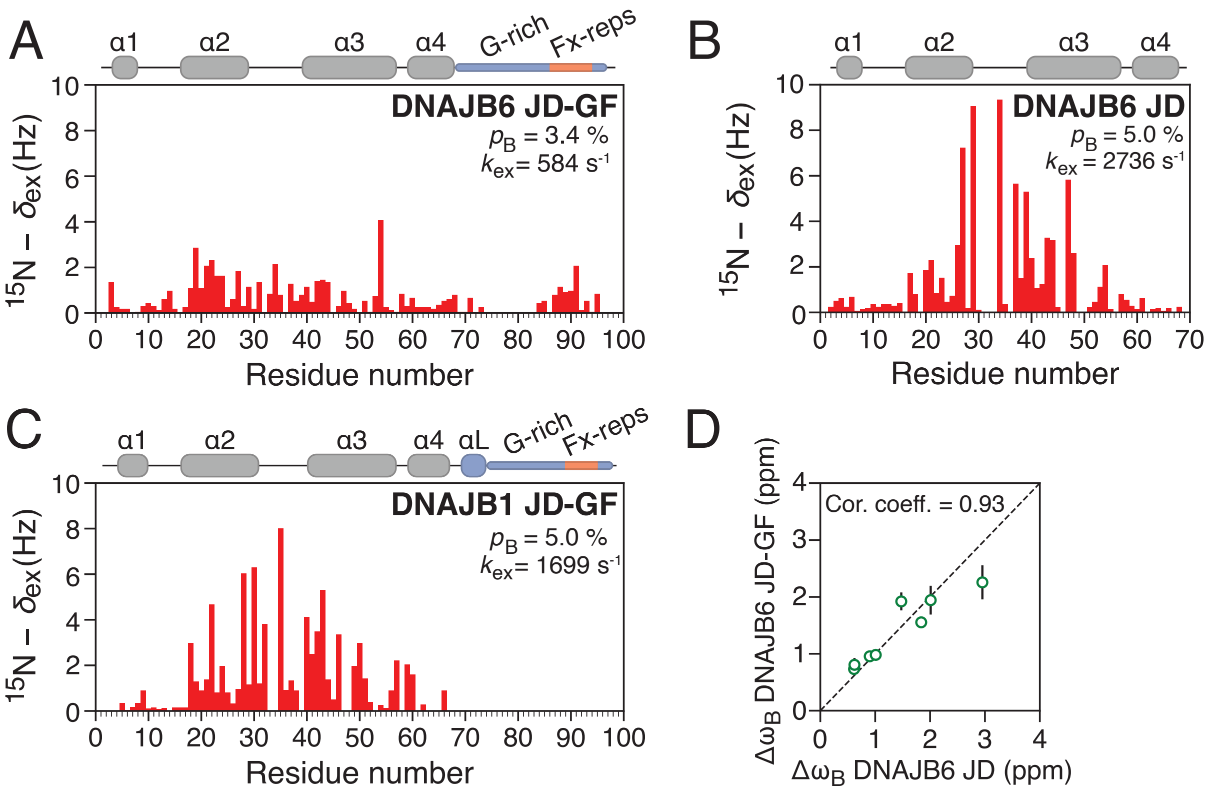

Supplementary Figure 9: Hsc70 binding to DNAJB6 and DNAJB1. ^{15}N-δ_{ex} values resulting from the binding of DNAJB6 JD-GF (A) or DNAJB6 JD (B) or DNAJB1 JD-GF (C) to full-length Hsc70. (D) Correlation between the fitted chemical shift values for DNAJB6 JD-GF and DNAJB6 JD. Error bars represent the errors calculated based on the covariance matrix of the fit to the CPMG and/δ_{ex} data.

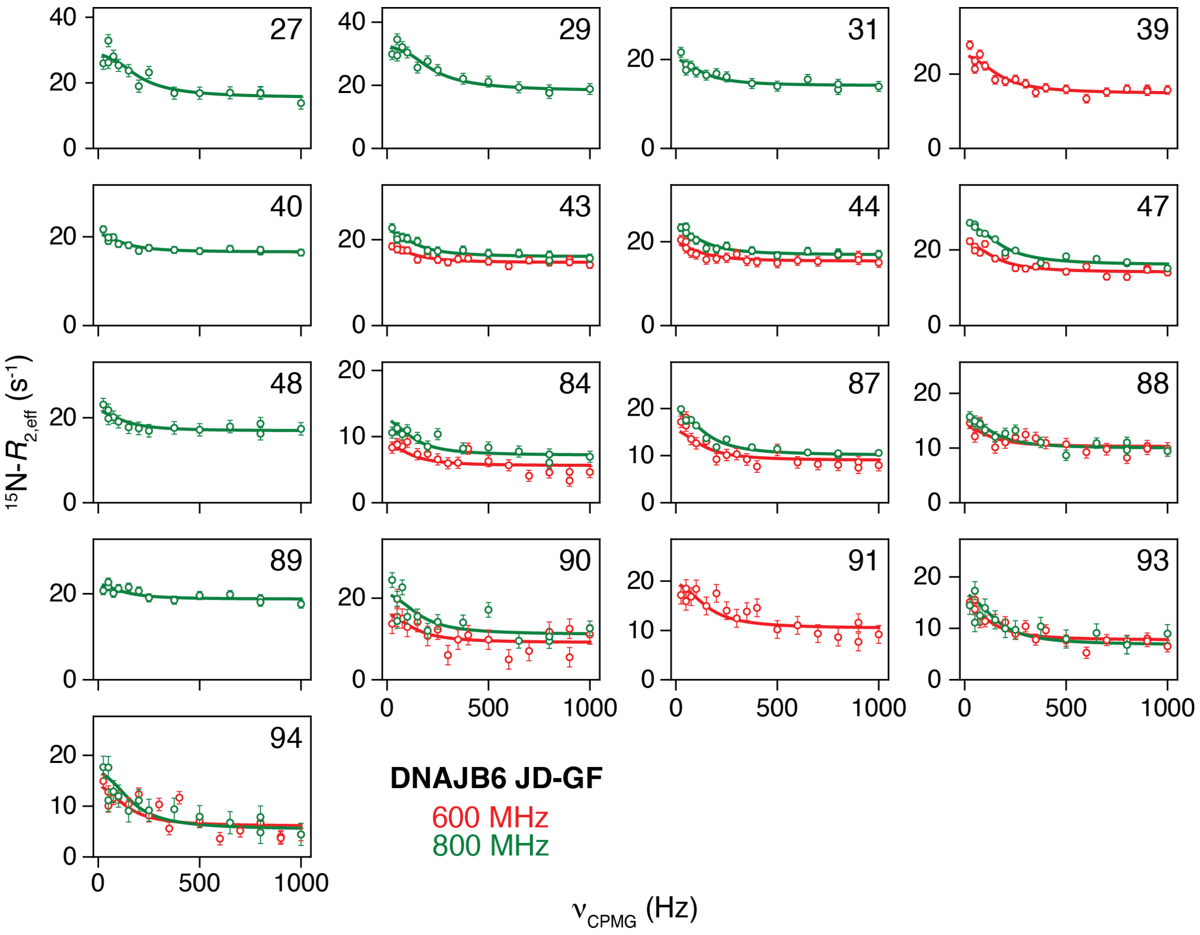

Supplementary Figure 10: Hsc70 binding to DNAJB6 JD-GF probed by ^{15}N-CPMG. In-phase ^{15}N-CPMG relaxation dispersion profiles of 200 μM NMR-visible DNAJB6 JD-GF in the presence of 20 μM unlabelled Hsc70 recorded at 800 (green) and 600 (red) MHz. The experimental data are displayed as circles, and the continuous lines represent best-fits to the two-state model of Eq. 1 and Fig. 6. Error bars are calculated based on duplicate measurements.

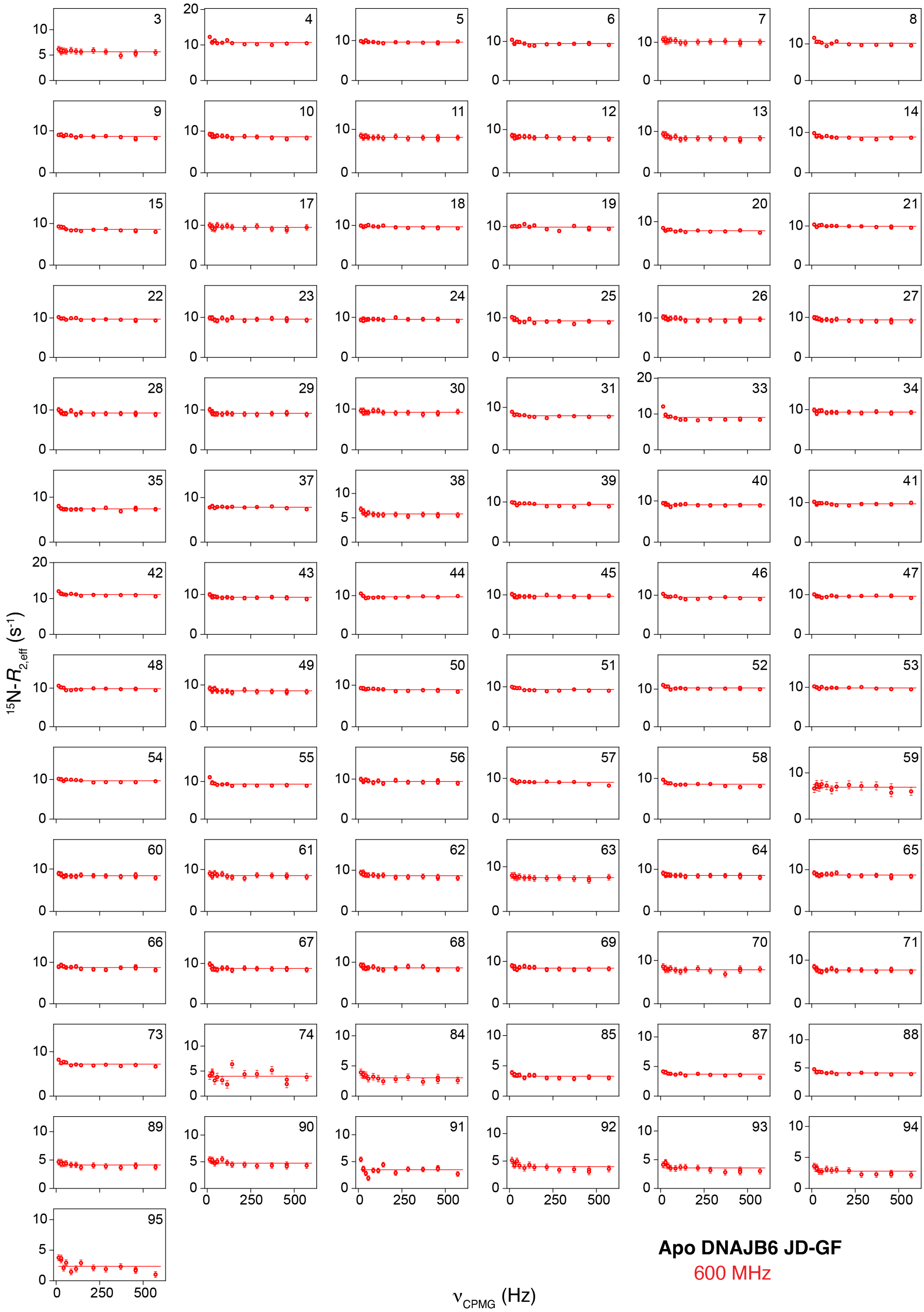

Supplementary Figure 11: Apo DNAJB6 JD-GF dynamics probed by ^{15}N-CPMG. In-phase ^{15}N-CPMG relaxation dispersion profiles of 300 µM DNAJB6 JD-GF recorded at 600 (red) MHz. Error bars are calculated based on duplicate measurements.

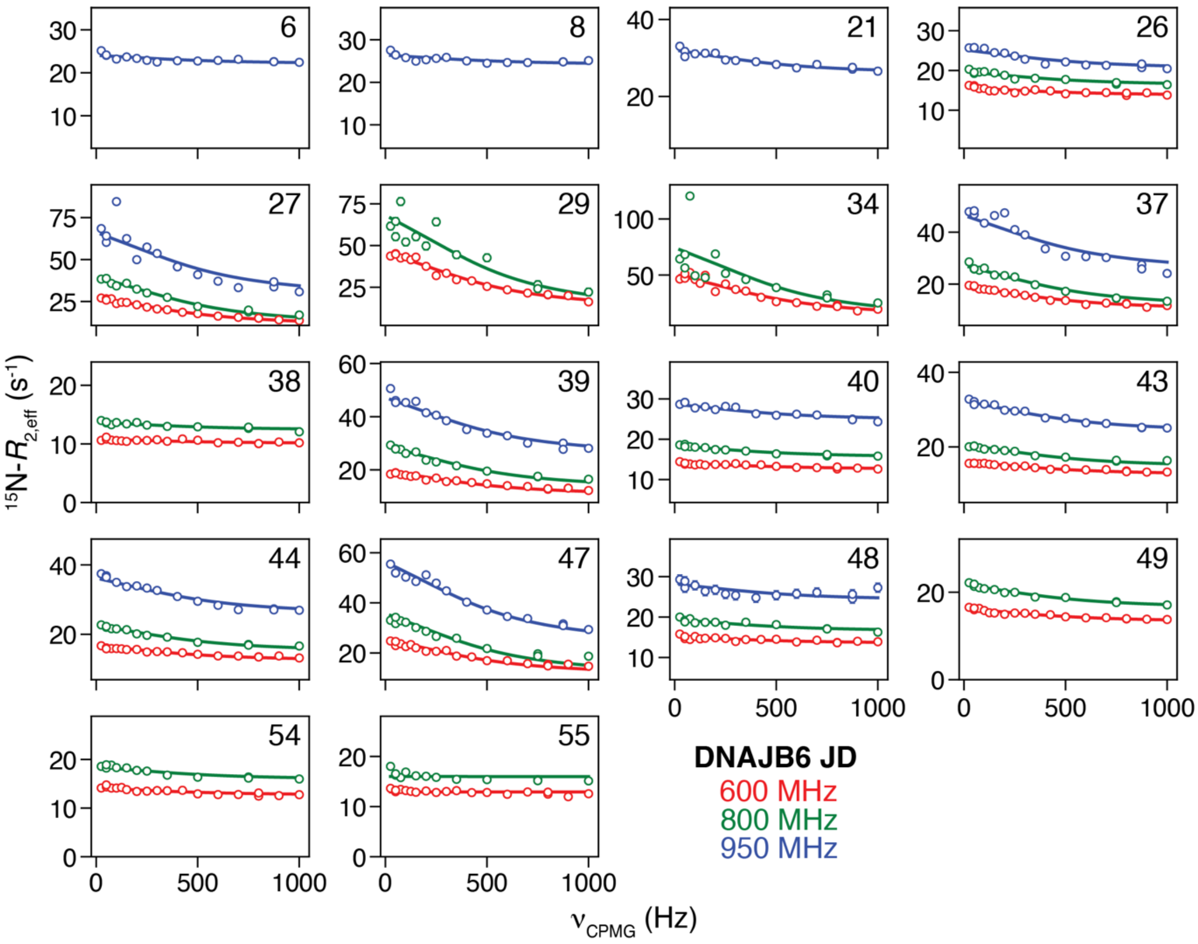

Supplementary Figure 12: Hsc70 binding to DNAJB6 JD probed by ^{15}N-CPMG. In-phase ^{15}N-CPMG relaxation dispersion profiles of 350 μM NMR-visible DNAJB6 JD in the presence of 35 μM unlabelled Hsc70 recorded at 950 (blue) 800 (green) and 600 (red) MHz. The experimental data are displayed as circles, and the continuous lines represent best-fits to the two-state model of Eq. 1 and Fig. 6. Error bars are calculated based on duplicate measurements.

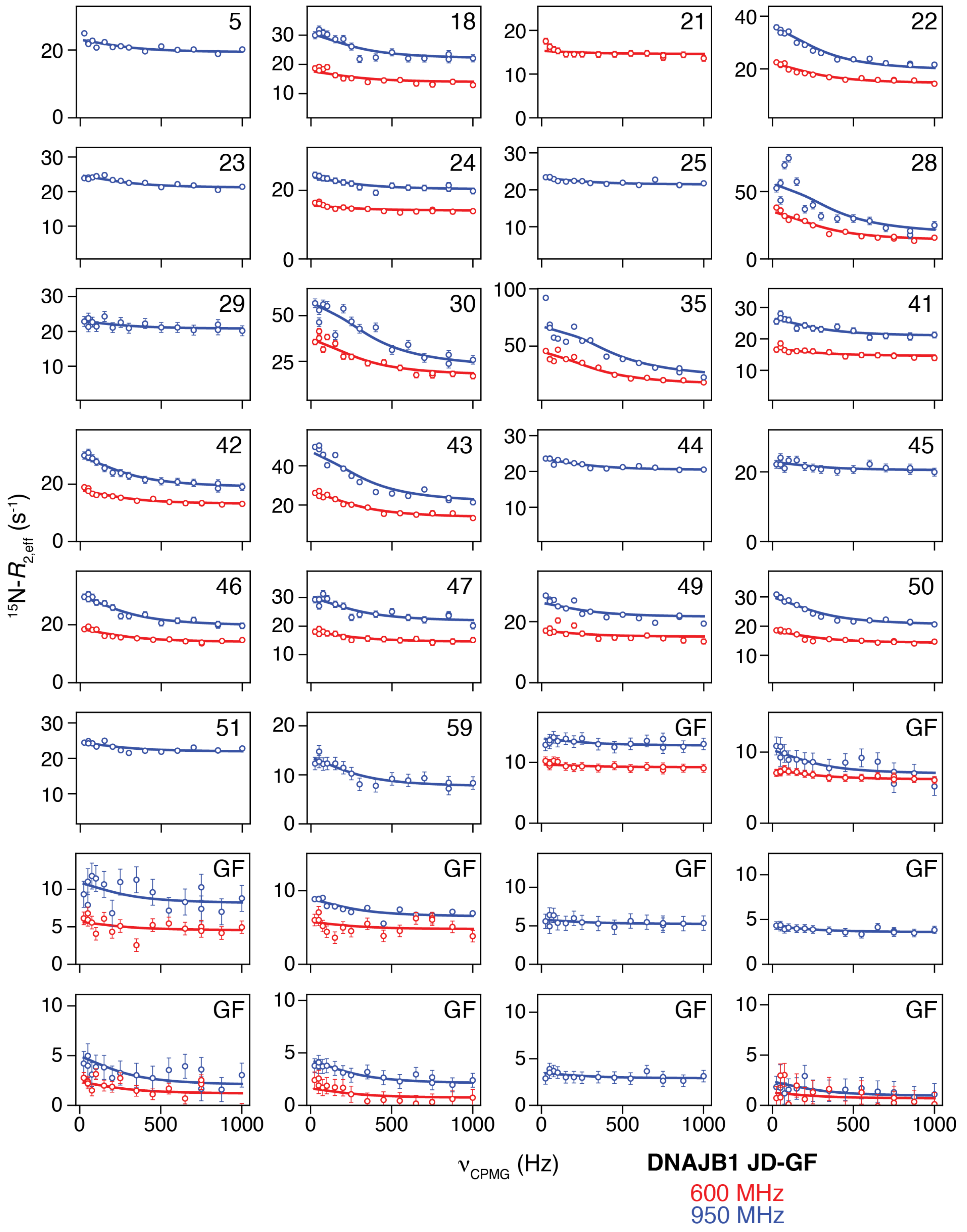

Supplementary Figure 13: Hsc70 binding to DNAJB1 JD-GF probed by ^{15}N-CPMG. In-phase ^{15}N-CPMG relaxation dispersion profiles of 300 μM NMR-visible DNAJB1 JD-GF in the presence of 30 μM unlabelled Hsc70 recorded at 950 (blue) and 600 (red) MHz. The experimental data are displayed as circles, and the continuous lines represent best-fits to the two-state model of Eq.1 and Fig. 6. Error bars are calculated based on duplicate measurements.

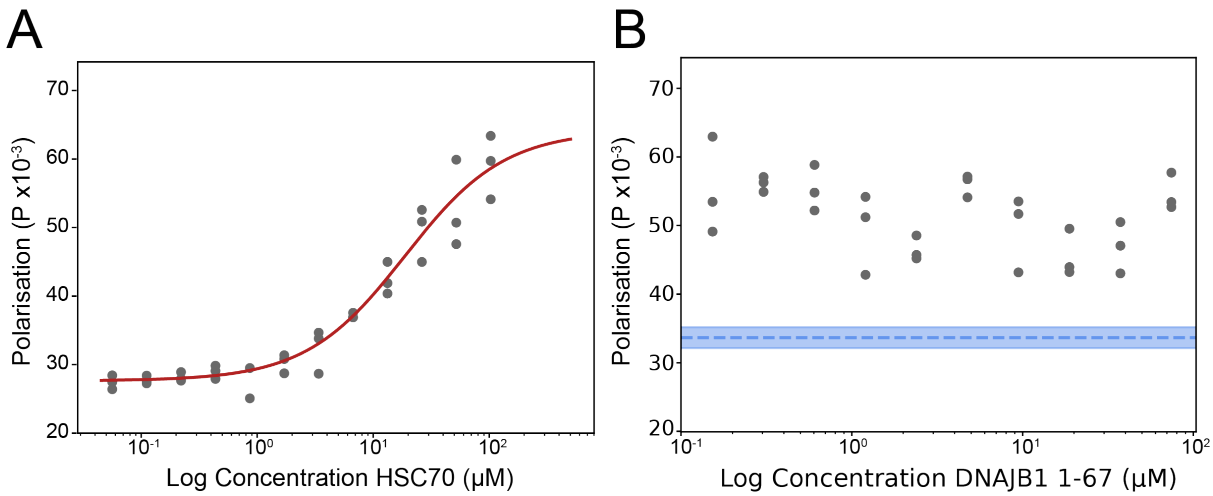

Supplementary Figure 14: JD binding to Hsc70 measured by fluorescence polarisation. (A) Titration of Hsc70 (T204A) into fluorescently labelled DNAJB6 JD (300 nM) in the presence of 5 mM ATP. Complex formation was followed by measuring fluorescence polarisation. Error bars show standard deviation for three technical repeats. Red line shows the fit to a four-point logistic curve, which yields a K_d of 18.0 ± 6.5 µM. (B) Competition experiment using DNAJB1 JD. Titration was performed using 300 nM fluorescently labelled DNAJB6 JD in the presence of 40 µM Hsc70 (T204A) and 5 mM ATP. The data show no significant drop in polarisation and hence no displacement of DNAJB6 up to the highest tested DNAJB1 JD concentration, 78.6 µM. Dotted blue line shows polarisation measured for free DNAJB6 JD in the absence of Hsc70, blue error band denotes standard deviation for this measurement. All measurements were performed as sets of 3 technical repeats which are shown as dots.

Supplementary Tables

Protein	k_{on} ($M^{-1}s^{-1}$)	k_{on}^{app} (s^{-1})	k_{off} (s^{-1})	k_{ex} (s^{-1})	K_d (μM)
DNAJB6 JD	$8.0\ (\pm 2.7) \times 10^8$	300 ± 6	2740 ± 51	3040 ± 60	3.4 ± 1.1
DNAJB6 JD-GF	$1.54\ (\pm 0.19) \times 10^6$	20.4 ± 1.8	580 ± 60	600 ± 60	380 ± 60
DNAJB1 JD-GF	$5.91\ (\pm 0.25) \times 10^6$	89.0 ± 2.1	1700 ± 40	1790 ± 40	288 ± 13
DNAJB1 JD	–	–	–	~ 2000	~ 450

Supplementary Table 1: Fitted kinetic parameters for the DNAJ – Hsc70 association. k_{on} and k_{on}^{app} are calculated as described in the Methods. The exchange rate k_{ex} is given by $k_{on}^{app} + k_{off}$ and $K_d = k_{off}/k_{on}$. The values given for DNAJB1 JD are based on preliminary fits only.

**(*a*) Sample details**	
Organism	*E.coli*
Source (Catalogue No. or reference)	
Description: sequence (including Uniprot ID + uncleaved tags), bound ligands/modifications, *etc.*	DNAJB6 JD, DNAJB6 JD-GF, DNAJB6 JD-GF-α5, DNAJB6 JD-GSS
Extinction coefficient ε (wavelength and units)	ε_{280}=14440 M^{-1} cm^{-1}
Partial specific volume $\bar{\upsilon}$ (cm^3 g^{-1})	0.7425 (for all samples)
Molecular mass *M* from chemical composition (Da)	DNAJB6 JD: 8230 Da<br>DNAJB6 JD-GF: 11085 Da<br>DNAJB6 JD-GF-α5: 12623 Da<br>DNAJB6 JD-GSS: 10512 Da
Concentration (range/values) measured and method	6/3/1.5/0.75 mg/ml (u.v. @ 280 nm)
Solvent composition and source	20 mM Sodium phoshate, 50 mM NaCl pH 7
**(*b*) SAS data collection parameters**	**Same for all samples**
Source, instrument and description or reference:	BioXolver L (Xenocs) equipped with a 250 W liquid gallium alloy X-ray source (metaljet)
Wavelength (Å).	1.34 Å
Beam geometry (size, sample-to-detector distance).	Size: 1*1 mm<br>Distance of 0.630 m
q-measurement range ($Å^{-1}$ or nm^{-1}).	0.015-0.51 ($Å^{-1}$)
Absolute scaling method.	Water standard
Method for monitoring radiation damage, X-ray dose where relevant.	Measurement of consecutive frames<br>(40 frames at 60 seconds each)
Sample configuration including path length and flow rate where relevant.	BioCube for sample delivery, no flow during exposure in a capillary.
Sample temperature (ºC).	22 ºC
**(*c*) Software employed for SAS data reduction, analysis and interpretation**	
SAS data reduction to sample–solvent scattering, and extrapolation, merging, desmearing *etc.* as relevant	Primary data processing performed in BioXTASRaw
Basic analyses: Guinier, *P*(*r*), scattering particle volume (*e.g.* Porod volume V_P or volume of correlation V_c)	ATSAS package V3.2.0-1
**(*d*) Structural parameters**	
**Guinier Analysis**	
I(0) (cm^{-1})	JD-GF: 0.02 (± 7.8x10^-5); JD: 0.02 (± 1.1x10^-4); JD-GF-α5: 0.03 (± 1.1x10^-4); JD-GSS: 0.03 (± 1.6x10^-4).
R_g (Å)	JD-GF: 17.3 ± 0.2; JD: 13.8 ± 0.1; JD-GF-α5: 15.7 ± 0.1; JD-GSS: 20.0 ± 0.1.
q-range ($Å^{-1}$)	JD-GF: 0.0237 - 0.0746; JD: 0.0159 - 0.0935; JD-GF-α5: 0.0280 - 0.0828; JD-GSS: 0.0180 - 0.0645.
Quality-of-fit parameter (with definition)	R^2 values of JD-GF: 0.99; JD: 0.96; JD-GF-α5: 0.99; JD-GSS: 0.92.

Supplementary Table 2. SAXS data acquisition and analysis. Details for SAXS data acquisition, samples and data analysis.

Supplementary References

1. Kroenke, C.D., Loria, J.P., Lee, L.K., Rance, M. & Palmer, A.G. Longitudinal and Transverse ^{1}H–^{15}N Dipolar/^{15}N Chemical shift anisotropy relaxation interference: unambiguous determination of rotational diffusion tensors and chemical exchange effects in biological macromolecules. *J. Am. Chem. Soc.* **120**, 7905-7915 (1998).
2. Fitzkee, N.C., Torchia, D.A. & Bax, A. Measuring rapid hydrogen exchange in the homodimeric 36 kDa HIV-1 integrase catalytic core domain. *Protein Sci.* **20**, 500-12 (2011).
